# Structure and Biological Roles of *Sinorhizobium fredii* HH103 Exopolysaccharide

**DOI:** 10.1371/journal.pone.0115391

**Published:** 2014-12-18

**Authors:** Dulce N. Rodríguez-Navarro, Miguel A. Rodríguez-Carvajal, Sebastián Acosta-Jurado, María J. Soto, Isabel Margaret, Juan C. Crespo-Rivas, Juan Sanjuan, Francisco Temprano, Antonio Gil-Serrano, José E. Ruiz-Sainz, José M. Vinardell

**Affiliations:** 1 IFAPA, Centro las Torres-Tomejil, Apartado Oficial 41200, Alcalá del Río, (Sevilla), Spain; 2 Departamento de Química Orgánica, Facultad de Química, Universidad de Sevilla, Sevilla, Spain; 3 Departamento de Microbiología, Facultad de Biología, Universidad de Sevilla, Sevilla, Spain; 4 Departamento de Microbiología del Suelo y Sistemas Simbióticos, Estación Experimental del Zaidín, CSIC, Granada, Spain; University Hospital of the Albert-Ludwigs-University Freiburg, Germany

## Abstract

Here we report that the structure of the *Sinorhizobium fredii* HH103 exopolysaccharide (EPS) is composed of glucose, galactose, glucuronic acid, pyruvic acid, in the ratios 5∶2∶2∶1 and is partially acetylated. A *S. fredii* HH103 *exoA* mutant (SVQ530), unable to produce EPS, not only forms nitrogen fixing nodules with soybean but also shows increased competitive capacity for nodule occupancy. Mutant SVQ530 is, however, less competitive to nodulate *Vigna unguiculata*. Biofilm formation was reduced in mutant SVQ530 but increased in an EPS overproducing mutant. Mutant SVQ530 was impaired in surface motility and showed higher osmosensitivity compared to its wild type strain in media containing 50 mM NaCl or 5% (w/v) sucrose. Neither *S. fredii* HH103 nor 41 other *S. fredii* strains were recognized by soybean lectin (SBL). *S. fredii* HH103 mutants affected in exopolysaccharides (EPS), lipopolysaccharides (LPS), cyclic glucans (CG) or capsular polysaccharides (KPS) were not significantly impaired in their soybean-root attachment capacity, suggesting that these surface polysaccharides might not be relevant in early attachment to soybean roots. These results also indicate that the molecular mechanisms involved in *S. fredii* attachment to soybean roots might be different to those operating in *Bradyrhizobium japonicum*.

## Introduction

Rhizobia are soil α- and β-proteobacteria that establish nitrogen-fixing symbioses with plants belonging to the *Leguminosae* family. As a result of this symbiotic interaction, a new plant organ, called the nodule, is developed on roots of leguminous plants where the bacteria fix nitrogen. Nodule development requires reciprocal molecular communication between the two symbiotic partners [1]–[3]. The specific flavonoid cocktail exuded by legume roots and the specific rhizobial lipochitooligosaccharides (also called Nod factors or LCOs) are two key-determinant signals that contribute significantly to the mutual recognition of the symbionts by acting at the very early stages of the nodulation process. Bacterial LCOs are necessary, but not sufficient, for the formation of nitrogen-fixing nodules on legume roots. In addition to nodulation (*nod, nol*, *noe*) genes and those involved in nitrogen fixation (*fix* and *nif* genes), other rhizobial signalling molecules are required for the formation of mature nitrogen-fixing nodules [4], [5]. Rhizobial surface polysaccharides, such as acidic exopolysaccharides (EPS), cyclic glucans (CG), lipopolysaccharides (LPS) and capsular polysaccharides (KPS) are clearly relevant for the formation of an effective symbiosis [6]–[13]. However, it is not yet known how a particular legume perceives compatibility factors other than LCOs.

In contrast to LPS and KPS, EPS is weakly associated with the bacterial surface and is released in large amounts into the cell's milieu [9]. The chemical structure of exopolysaccharides produced by diverse rhizobial species has been determined. They typically consist of branched repeating units formed by a variable number (from 2 to 9) of monosaccharides [14], [15]. Glucose is the most abundant monosaccharide of rhizobial EPS, with the exception of the EPS produced by *Bradyrhizobium japonicum*, *Azorhizobium caulinodans*, and *Rhizobium* sp. isolated from *Vigna mungo* nodules [14]. Sugar composition and their linkage in the repeating unit, repeating unit size and their degree of polymerization as well as non-carbohydrate decoration account for the large diversity of EPS structures found among rhizobia [14]. *Sinorhizobium meliloti* Rm1021 produces two exopolysaccharides that are structurally distinct. EPS I (also called succinoglycan) contains an octasaccharidic repeating unit composed of glucose and galactose at a 7∶1 molar ratio [16], while the repeating unit of EPS II (the so-called galactoglucan) is a disaccharide of glucose and galactose [17]. The presence of acetyl, succinyl, and pyruvic acid (as ketal) substituents confers anionic properties to EPS [16].

Genes involved in EPS I synthesis (*exo*) have been extensively studied and many fundamental aspects of the EPS I biosynthesis pathway are well known [15], [18]–[20]. Similarly to other bacterial EPS, rhizobial EPS play a significant role in biofilm formation, being the major component of its matrix, which provides a physical barrier against diffusion of toxic compounds and protection against environmental stresses [21].

Different reports set forth the notion that EPS is, in general, necessary for the infection and correct formation of indeterminate nodules but not very relevant for determinate-nodule-forming symbioses [18], [22], [23]. For example, in *S. meliloti* Rm1021 and *Rhizobium leguminosarum* bv. *trifolii* an increased production of EPS I has been described to enhance symbiosis with *Medicago truncatula* and *Trifolium pratense* respectively [24], [25]. The species *Rhizobium leguminosarum,* which includes three biovars, is an interesting example of this issue. Thus, *R. leguminosarum* biovars *trifolii* and *viciae*, which infect plants forming indeterminate nodules, require EPS for successful symbiosis, whereas biovar *phaseoli*, which nodulates *Phaseolus* (determinate nodules) does not [25]–[30]. However, the situation is actually more complicated and appears to depend more on the specific bacterium-host plant relation. For example, *S. fredii* HH103 EPS is not required for effective symbiosis with *Glycyrrhiza uralensis*, a legume which forms indeterminate nodules [31], and *Bradyrhizobium japonicum exoB* mutants not only showed delayed nodulation and severe loss of their competitive capacity in soybean, but also induced the occurrence of plant defence reactions [32], [33].

*Sinorhizobium fredii* HH103 is a fast growing rhizobial strain that nodulates *Glycine max* (soybean) and many different herbaceous, shrub and tree legumes able to form determinate or indeterminate nodules [34]. The genome sequence of *S. fredii* HH103 (one chromosome and 6 plasmids) is available in the EMBL Nucleotide Sequence Database (EMBL-Bank) under accession numbers HE616890 to HE616899 [35]. *S. fredii* HH103 produces at least five different surface polysaccharides: exopolysaccharides (EPS), lipopolysaccharides (LPS), two different types of capsular polysaccharides (KPS [K-antigen polysaccharides]), and cyclic glucans (CG). *S. fredii* HH103 CG consists of 18–24 units of →2)-β-d-Glc*p*-(1→, partially substituted with glycerol-1-phosphate at the C-6 position of some of the glucose units [7]. Two different types of KPS are constitutively produced by *S. fredii* HH103. One of them, called poly-PseAc, is a homopolymer of a derivative of the pseudaminic acid [36], while the other is a homopolymer of 3-deoxy-D-manno-oct-2-ulosonic acid [37] for which no symbiotic role has been assigned.

*S. fredii* HH103 mutants affected in the production of KPS, CG or EPS have been already constructed and described. HH103 mutants unable to produce EPS are fully effective with soybeans [11], while those unable to produce CG only form small knot like structures (pseudonodules) that do not fix nitrogen and are devoid of rhizobial cells [7]. The HH103 poly-PseAc (hereafter called “KPS”) plays an important role in the *S. fredii*-soybean symbiosis, since mutants affected in genes of the *rkp-1* and *rkp-3* regions are symbiotically impaired with soybean [11], [31], [38], [39]. All these studies led to the conclusion that KPS and GC, but not EPS, are relevant for the symbiotic capacity of *S. fredii* HH103 with soybean. In this work we determine the chemical structure of the *S. fredii* HH103 EPS and show that a *S. fredii* HH103 EPS mutant is more competitive than its parental strain to nodulate soybean cv. Williams. In *Vigna unguiculata* plants, however, this HH103 EPS mutant is outcompeted by its parental wild-type strain. We also conclude that *S. fredii* HH103 attaches to soybean roots by a mechanism in which neither the bacterial polysaccharides mentioned above nor soybean lectin (SBL) appear to be involved. Furthermore, we show that HH103 EPS is essential for the capacity of this strain to cope with an osmotic stress and to form biofilms in both plastic and glass surfaces, and that this polysaccharide has also a role in surface motility.

## Results

### Determination of the chemical structure of the *S. fredii* HH103 exopolysaccharide

The exopolysaccharide (EPS) was isolated from culture medium of *S. fredii* HH103 by precipitation with ethanol and then purified by dialysis. Proteins had been previously eliminated by treatment with proteinase K. Initial composition analysis indicated that EPS was composed by glucose and galactose in a ratio close to 5∶2.

**Figure 1:**
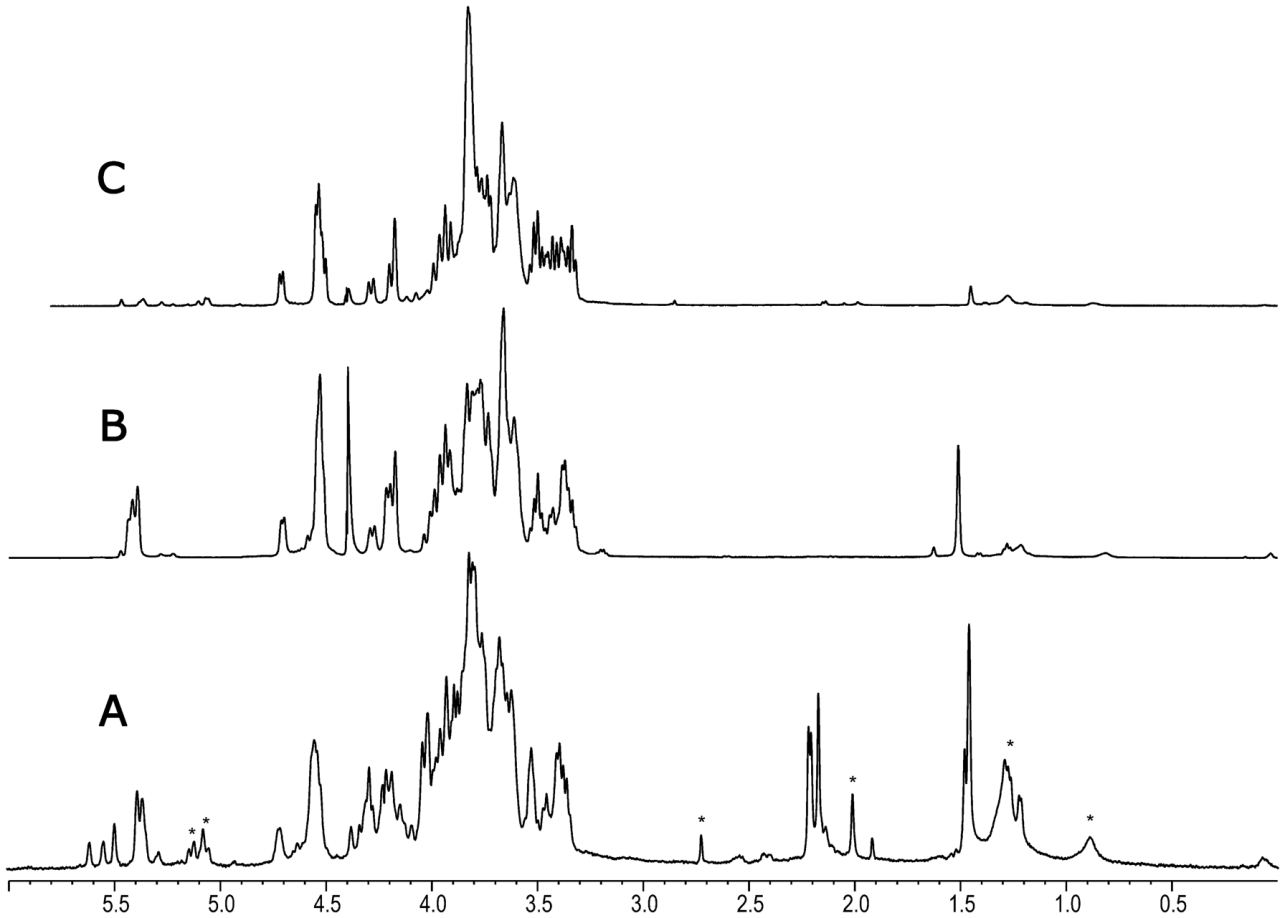
^1^H-NMR (500 MHz) spectra of exopolysaccharide of *S*. *fredii* HH103: a) after isolation and purification by dialysis, b) after partial hydrolysis, and c) after lithium degradation. Signals from culture medium are marked with asterisks.

^1^H-NMR spectrum (Fig. 1A) shows multiple signals in the anomeric region (4.50 to 5.7 ppm) and signals of possible substituent groups: acetyl (close to 2.2 ppm) and pyruvate (close to 1.5 ppm); given the complexity of the structure, the initial approach was to partially hydrolyse the polysaccharide and to study the structure of the isolated oligosaccharides. Thus, EPS was treated with 0.5M TFA at 100°C and the resulting oligosaccharides were isolated by dialysis, size exclusion chromatography (SEC), and silica gel chromatography. NMR studies on one of the fractions (S1 Figure) indicates that it contains the trisaccharide α-Glc*p*A-(1→3)-α-Glc*p*A-(1→4)-Glc. The carboxyl group of uronic acids stabilizes glycosidic linkages during hydrolysis [40], which explains that glucuronic acid had not been found in the monosaccharide analysis. Additionally, NMR studies on another fraction (data not shown) indicates the presence of a →4)-α-Glc*p*A and a non-reducing terminal α-Gal*p* substituted at *O*-4 and *O*-6 by a pyruvate group as ketal.

Carboxylic groups of uronic acids were reduced to –CD_2_OH groups by the method of carbodiimide (using NaBD_4_) and the modified polysaccharide was submitted to several analyses. The monosaccharide analysis indicates that it contains glucose and galactose in a ratio 7∶2. The absolute configuration analysis showed that all of the residues are d. Finally, methylation analysis gave the partially methylated and acetylated alditols corresponding to →3)-d-Gal*p*, →3)-d-6-*d*_2_-Glc*p* (from a glucuronic acid unit), →4)-d-Glc*p* (coeluting with →4)-d-6-*d*_2_-Glc*p*), →6)-d-Glc*p*, →4,6)-d-Glc*p*, and →4,6)-d-Gal*p* in a ratio close to 1∶1∶4∶1∶1∶1.

The occurrence of →3) and →4)-linked uronic acids makes appropriate the degradation of EPS with lithium in ethylenediamine [41]. When the exopolysaccharide was treated in such way, a polysaccharide was obtained (EPS-Li), which was purified by SEC and studied by NMR; this result indicates that glucuronic acid units are located in branches, and not in the main chain.

**Figure 2:**
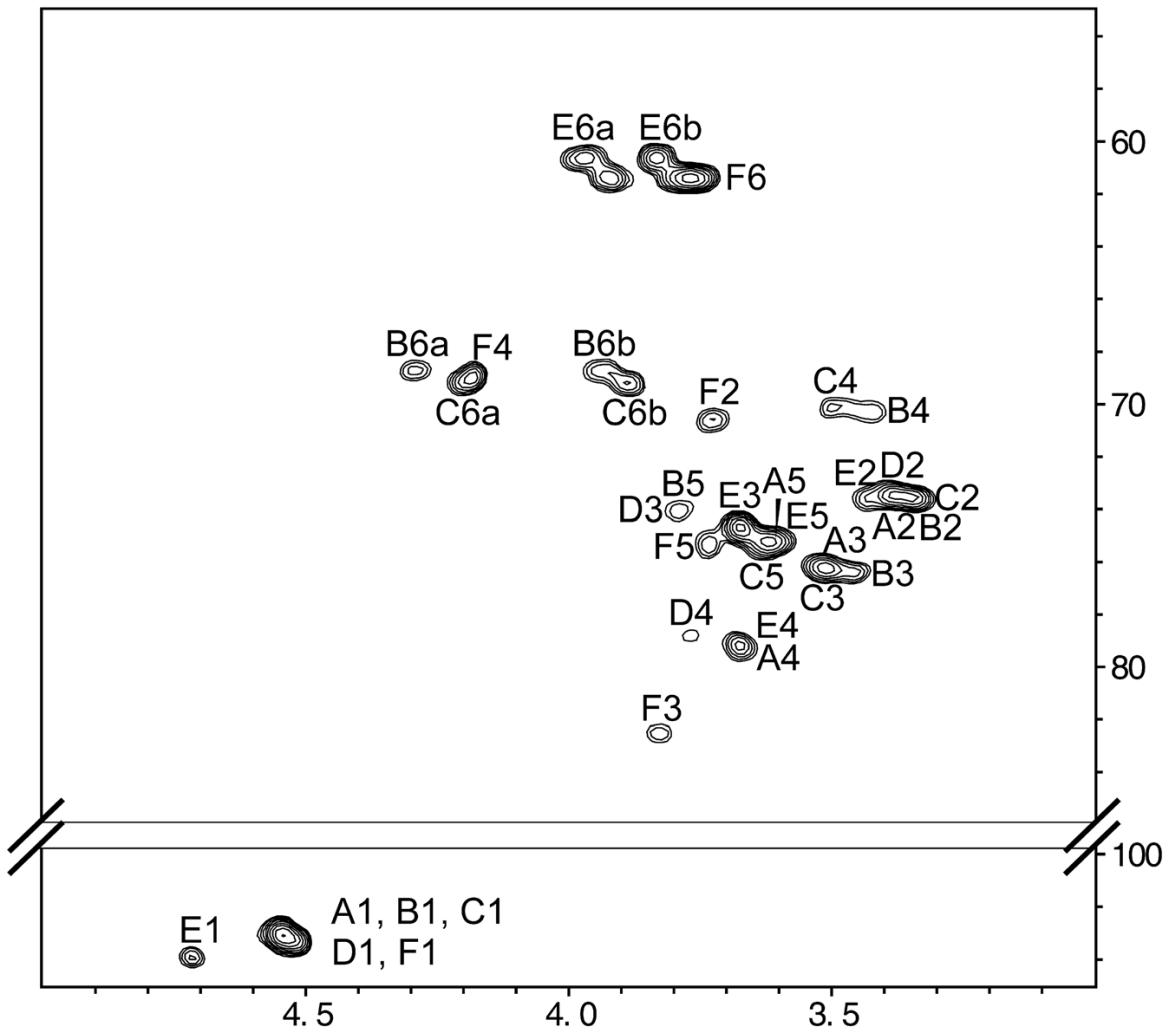
^1^H (500 MHz)-^13^C (125 MHz) HSQC of the lithium-degraded polysaccharide obtained from EPS.

**Figure 3:**
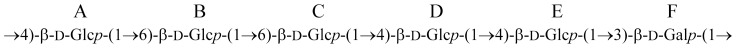
Structure for the lithium-degraded polysaccharide obtained from EPS.

^1^H-NMR of EPS-Li (Fig. 1C) shows only signals in the anomeric region corresponding to β-configurations. 2D NMR experiments (DQF-COSY, TOCSY, ROESY, HSQC (Fig. 2), HMBC, and HMQC-TOCSY), together with data from program CASPER [42] lead to the determination of the structure for this polysaccharide, as presented in Fig. 3 and summarized in S1 Table. Thus, EPS-Li consists of an unbranched polysaccharide with a repeating unit containing five glucose and one galactose units, all of them having β-configurations. Moreover, according with the previous monosaccharide and methylation analysis, the exopolysaccharide repeating unit must have a branch composed of a galactose and two glucuronic acid residues.

**Figure 4:**
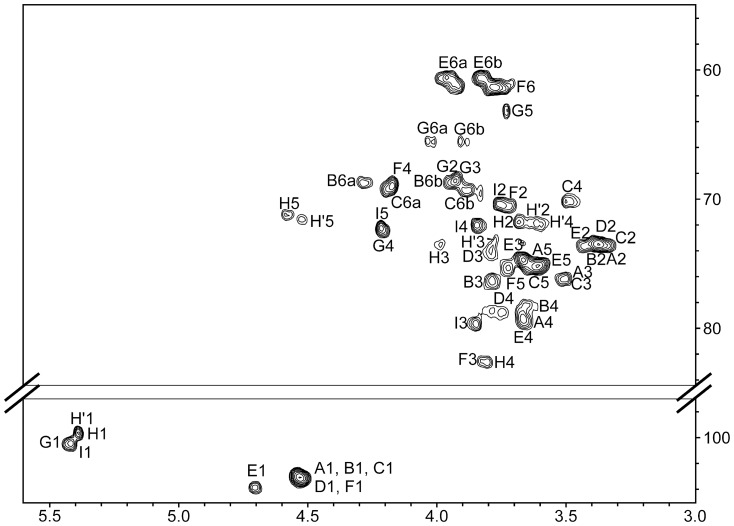
^1^H (500 MHz)-^13^C (125 MHz) HSQC of the partially hydrolysed polysaccharide obtained from EPS.

The study of the complete sugar backbone was carried out on the polysaccharide obtained after partial hydrolysis and purified by SEC. Its ^1^H-NMR spectrum is shown in Fig. 1B. It is significant the presence of signals at 5.4 ppm, corresponding to α-anomeric protons, and 1.5 ppm, characteristic of an pyruvate ketal group. Again, 2D NMR experiments (DQF-COSY, TOCSY, HSQC (Fig. 4), and HMBC) as well as the NMR study of EPS-Li allow the assignment of most of the NMR signals (Table 1). Signals at 5.4 ppm corresponds to three residues with α-configuration, forming the oligosaccharide α-d-Gal*p*-(1→4)-α-d-Glc*p*A-(1→3)-α-d-Glc*p*A-(1→; the galactose residue is substituted at *O*-4 and *O*-6 with a pyruvate ketal group, whose absolute configuration can be deduced from chemical shifts of residue G [43]. The branching point can be identified by a downfield shift of C-4 of residue B with regard to its value in EPS-Li (78.3 and 70.3 ppm, respectively). The hydrolysis has partially released the terminal galactose residues in this polysaccharide, as they can be found signals corresponding to a terminal glucuronic acid unit (labelled H′). This structure has the same carbohydrate backbone than that found in the exopolysaccharide isolated from *Sinorhizobium fredii* strain NGR234 [44]–[46]. When comparing ^1^H-NMR spectra of exopolysaccharide isolated from *S*. *fredii* HH103 (Fig. 1A) and *S. fredii* strain NGR234 [47] it can be seen that both spectra are almost identical. It is necessary to point out that both GlcA residues are α in the structure described for NGR234 by Djordjevic et al. [44], but it was changed by mistake in one of the figures of a later publication in spite of being correctly described in the text. Unfortunately, this mistaken figure has been taken in later publications.

**Table 1:** Chemical shifts (^1^H and ^13^C) for the partially hydrolysed exopolysaccharide isolated from *S*. *fredii* HH103.

Unit	Signal	1	2	3	4	5	6a	6b
A	^1^H	4.55	3.36	3.51	3.67	3.61	n.a^a^	n.a
→4)-β-d-Glc*p*	^13^C	103.0	73.5	76.2	79.3	75.3	n.a	
B	^1^H	4.52	3.34	3.79	3.65	3.78	4.29	3.94
→6)-β-d-Glc*p*	^13^C	103.2	73.6	76.3	78.3	74.0	68.7	
C	^1^H	4.53	3.34	3.50	3.50	3.62	4.19	3.88
→6)-β-d-Glc*p*	^13^C	103.1	73.6	76.2	70.1	75.3	69.2	
D	^1^H	4.54	3.38	3.79	3.75	n.a	n.a	n.a
→4)-β-d-Glc*p*	^13^C	102.9	73.4	74.1	78.8	n.a	n.a	
E	^1^H	4.71	3.43	3.67	3.63	3.60	3.95	3.83
→4)-β-d-Glc*p*	^13^C	103.9	73.6	74.7	79.2	75.1	60.6	
F	^1^H	4.54	3.72	3.83	4.17	3.72	3.77	3.77
→3)-β-d-Gal*p*	^13^C	103.1	70.6	82.5	68.9	75.3	61.4	
G	^1^H	5.44	3.93	3.93	4.22	3.73	4.03	3.89
→4,6)-α-d-Gal*p*	^13^C	100.5	68.6	68.5	72.2	63.1	65.5	
H	^1^H	5.39	3.68	3.98	3.81	4.58	_	
→4)-α-d-Glc*p*A	^13^C	99.7	71.8	73.6	82.6	71.2	n.a	
H′	^1^H	5.39	3.63	3.77	3.59	4.52	_	
α-d-Glc*p*A	^13^C	99.6	71.9	73.3	71.9	71.6	n.a	
I	^1^H	5.41	3.75	3.85	3.84	4.21	_	
→3)-α-d-Glc*p*A	^13^C	100.5	70.4	79.7	72.0	72.4	n.a	
*R*-Pyr	^1^H	_	_	1.51				
	^13^C	174.5	100.0	25.5				

a Not assigned.

The exopolysaccharide of NGR234 bears up to three *O*-acetyl groups, two of them located on the non-reducing terminal galactose (*O*-2 and/or *O*-3) and a third one whose location has not been determined [45]. NMR of exopolysaccharide from *S*. *fredii* HH103 shows signals from three acetyl groups (Fig. 1A); two of them are quite labile as they are lost after heating in water at 80°C for several hours (S2 Figure). Regarding H-1 of unit G, it appears as three different signals in a ratio 50∶25∶25, corresponding to an α-Gal*p* residue without acetyl groups (G1), or with H-1 downfield shifted because of neighbour acetyl groups at *O*-2 and/or *O*-3. After heating, acetyl groups are released, as signals from G1′ and G1″ decrease together with those from two acetyl groups (Ac1 and Ac2). A third acetyl group remains unchanged under this treatment and its location has not been determined.

**Figure 5:**
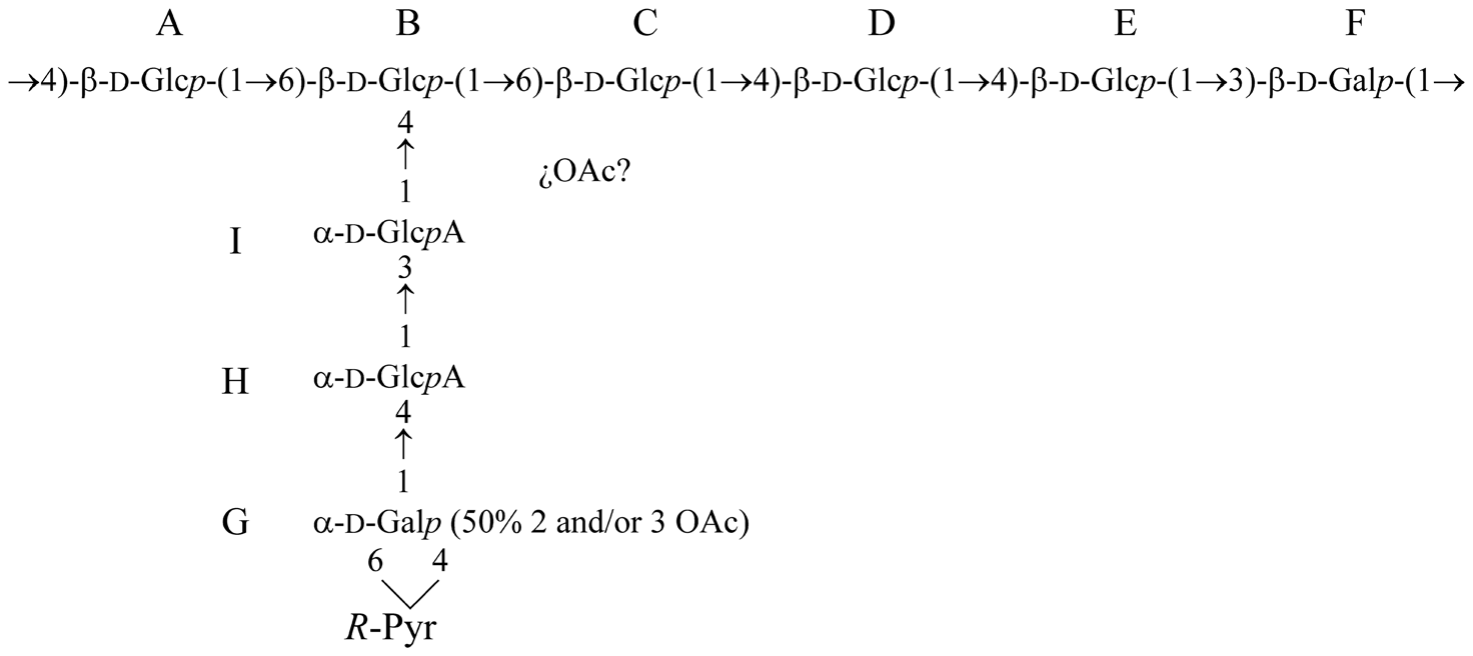
Structure for the exopolysaccharide isolated from *S. fredii* HH103. The structure includes three *O*-acetyl groups, two of them located at *O*-2 and/or *O*-3 of unit G. The location of the third acetyl group is unknown.

All these results show that the chemical structure of the EPS produced by *S. fredii* HH103 is that described in Fig. 5, being equal to that of *S. fredii* NGR234 [44].

### The *S. fredii* HH103 *exo* cluster

**Figure 6:**
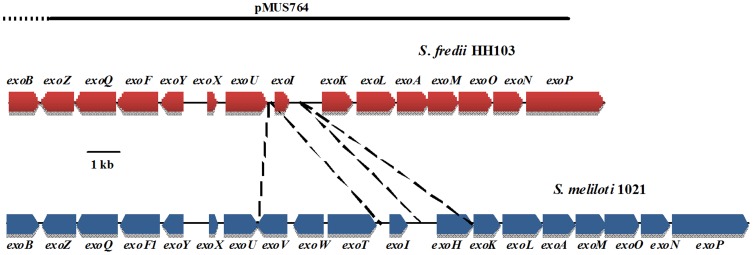
Genetic organization of the *S. fredii* HH103 *exo* region and a comparison to that of *S. meliloti* 1021. The HH103 DNA region covered by cosmid pMUS764 is also shown.

The *S. fredii* HH103 genome sequence has become available recently [34], [35]. Most genes involved in EPS biosynthesis are located on the largest plasmid (pSfHH103e, 2096125-bp, Accession: NC_016815.1) and grouped into a cluster that present the same genetic organisation than that found in *S. fredii* NGR234. This cluster (Fig. 6) includes a regulatory gene (*exoX*), genes involved in EPS polymerisation and transport (*exoF*, *exoP*, *exoQ*, *exoK*), as well as structural genes coding for glucosyl transferases (*exoA*, *exoL*, *exoM*, *exoO*, *exoU*), a galactosyl transferase (*exoY*), an UDP-glucose-4-epimerase (*exoB*), an acetyl transferase (*exoZ*), and *exoN* (which codes for the protein responsible for the synthesis of UDP-glucose from glucose-1P). This genetic region is also very similar to that present in *S. meliloti* Rm1021, although the later contains 4 genes that are not present in *S. fredii* HH103: *exoV* (coding for a pyruvyl transferase), *exoW* (glycosyl transferase), *exoT* (Wzx-type transport protein), and *exoH* (succinyl transferase).

In a previous work we obtained a *S. fredii* HH103 mutant derivative, called SVQ530, in the *exoA* gene [11]. NMR analyses confirmed that the extracellular milieu of *S. fredii* SVQ530 cultures does not contain EPS (our own unpublished results). This is what it is expected if the ExoA protein has the same function in *S. fredii* HH103 and *S. meliloti* Rm1021: the addition of the first glucose (Fig. 5, E) to the lipid-galactose structure (Fig. 5, F) of the nascent EPS repeating unit.

### Competition for nodulation of a *S. fredii* HH103 mutant (*exoA*) unable to produce exopolysaccharide

The *S. fredii* HH103 *exoA* mutant, SVQ530, induces the formation of nitrogen fixing nodules in both *Glycine max* cv. Williams and *Vigna unguiculata* cv. Bisbee Red [11], [38]. S3 Figure shows the aspect of both roots and aerial parts of plants inoculated with strain SVQ530 or its parental strain HH103 Rif^R^. SVQ530 not only forms nitrogen fixing nodules with soybean Williams [11] but also with the wild soybean (*Glycine soja*) accession PI597455 (data not shown). Since the mutation in *exoA* did not impair *S. fredii* HH103 to nodulate primitive and advanced soybeans, four independent competition assays were carried out to investigate the capacity of SVQ530 to occupy soybean Williams nodules in competition with its parental wild type strain HH103. The percentage of soybean nodules occupied by the gentamycin-resistant co-inoculant (SVQ530) was higher in all the four experiments and significantly different (p<0.05) in three of them (Table 2). These results suggest that the absence of EPS production enhances, or at least does not affect negatively, the *S. fredii* HH103 capacity to compete for soybean nodule occupancy. In *Vigna unguiculata*, however, mutant SVQ530 was significantly less competitive than its parental EPS-producing strain (Table 2).

**Table 2:** Competition between HH103 Rif^R^ and its *exoA* derivative (SVQ530) on *Glycine max* cv. Williams and *V. unguiculata* cv. Bisbee Red.

Legume	Experiment	Total number of nodules analyzed	% nodules occupied by SVQ530
*Glycine max*	I	161	60.8 **
	II	96	56.2
	III	120	77.5 **
	IV	120	62.0 **
*Vigna unguiculata*	I	120	19.2 **
	II	120	41.6 *

Nodule occupancy by strain SVQ530 was determined by assessing the gentamycin resistant marker (presence of the *lacZ*Δp-Gm^R^ cassette). Determinations were carried out 6 weeks after inoculation. Level of significance (p<0.05 ** and p<0.10 *) of the Chi-square analyses for nodule occupancy.

### *S. fredii* HH103 does not bind to soybean lectin (SBL)

**Figure 7:**
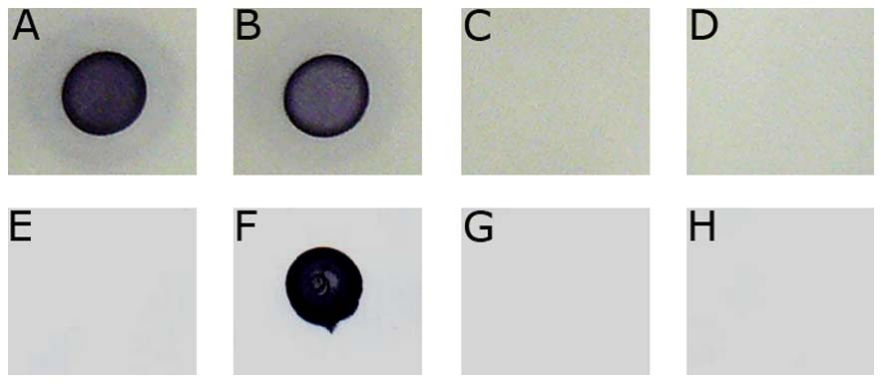
Soybean-lectin (SBL) binding to *Bradyrhizobium* and *S. fredii* strains. SBL was labelled with peroxidase as described by Liang and Emerich (1987). Panels: **A**, **B**: *B. japonicum* USDA110; **C**, **D**: *S. fredii* HH103; **E**: *B. elkanii* USDA46; F: *B. liaoningense* 2281; **G**: *S. fredii* SMH12; **H**: *S. fredii* HWG35. Panels B and D correspond to cultures grown in the presence of 3.37 µM genistein.

The fact that mutant SVQ530 is not negatively affected in its competitive capacity to nodulate soybean prompted us to investigate whether *S. fredii* HH103 binds, or not, to soybean lectin (SBL). Lectin-binding capacity of different fast- and slow-growing soybean rhizobia grown on nitrocellulose filters was determined using peroxidase-labelled soybean lectin (Sigma) as described previously [48]. Lectin binding was considered positive if the area where the bacteria grew became blue (Fig. 7). All the *B. japonicum* strains tested showed positive binding to the peroxidase-labelled soybean lectin while those belonging to *B. elkanii* did not develop any detectable blue colour. Positive signals were also found with *B. liaoningense* (another soybean symbiont). Forty-two *S. fredii* strains (including HH103) and other *Bradyrhizobium* strains that do not nodulate soybeans also failed to bind the SBL (Table 3).

**Table 3:** Soybean lectin (SBL) binding assays to different species of *Bradyrhizobium* and *Sinorhizobium*.

Bacteria	Strain	Binding to soybean lectin (SBL) conjugated with peroxidase A	Symbiotic capacity with *Glycine max* B
***Bradyhizobium***			
*B. japonicum*	USDA6, USDA38, USDA110, USDA123, USDA122, USDA136 ( = CB1809), USDA138.	+	Fix^+^
*B. elkanii*	USDA46, USDA76	-	Fix^+^
*Bradyrhizobium liaoningense*	2281	+	Fix^+^
*B. betae*	PL7HG1	-	Nod^- (**C**)^
*B. pachyrhizi*	PAC48	-	Nod^- (**D**)^
*B. canariense*	ISLU16	-	Nod^-^
***Sinorhizobium fredii***			
- Bacteria isolated from Chinese soils	USDA192, B1, B2, B4, B6, B8, B50, HH4, HH18, WH5, WH7, HW26, S4	-	Fix^+ (**E**)^
- Bacteria isolated from Chinese soils	HH103, B33, HH3, HH5, HH25, HH29, HHG35, WH4, WHG11, WHG14-S, HW1, HW5, HW16, HW22, HWG35, WW2, WW9, WWG11, WWG14, S1, S2, S5, S8, S28, S44, S48, S49	-	Fix^+ (**F**)^
- Strain isolated from Vietnamese soils	SMH12	-	Fix^+ (**F**)^
- Strain isolated from Papua New Guinean soils	NGR234	-	Nod^- (**G**)^

A +, dark purple spot; -, absence of coloured spot.

B Nod^-^, nodules are not formed; Fix^-^, ineffective or poorly effective nodules are formed; Fix^+^, effective nitrogen-fixing nodules are formed.

C Bacteria isolated from *Beta vulgaris* (sugar beet).

D Bacteria isolated from *Pachyrhizus ahipa*.

E Fix^+^ with some Asiatic soybean cultivars but Nod^-^ or Fix^-^ with the American soybean cv. Williams.

F Fix^+^ with some Asiatic soybean cultivars and also with the American soybean cv. Williams.

G Broad host range *S. fredii* strain isolated from nodules of *Lablab purpureus*. Nod^-^ with all soybeans tested.

*S. fredii* HH103 Rif^R^ cultures grown in the presence of the flavonoid genistein did not bind to SBL (Fig. 7D), which indicates that this soybean-secreted flavonoid able to induce the transcription of *S. fredii* HH103 nodulation genes [49] does not induce the presence of SBL receptors.

### *S. fredii* HH103 mutants affected in bacterial surface polysaccharides (SPs) are not significantly impaired in their attachment capacity with soybean roots

Because mutant SVQ530 showed enhanced competitiveness to nodulate soybeans in spite of not producing EPS, we investigated whether the capacity of this mutant to attach to soybean roots was different to that of its parental-strain HH103 Rif^R^. Since rhizobial mutants impaired in the production of a particular surface polysaccharide (SP) can be also affected in the production of other SPs, a collection of *S. fredii* HH103 mutants negatively affected in the production of diverse SPs (EPS, cyclic glucans, KPS and LPS) were also included in this study. None of the *S. fredii* HH103 SPs mutants showed any clear reduction of the number of bacteria attached to soybean cv. Williams roots (Table 4). Thus, in the experimental conditions used, *S. fredii* HH103 surface polysaccharides (SPs) appear not to be significantly involved in attachment to soybean roots.

**Table 4:** Binding assays of *S. fredii* HH103 Rif^R^ and different surface polysaccharide (SPs) mutants to *Glycine max* cv. Williams roots.

Inoculant A	Characteristics of the inoculants	Number of bacteria attached per gram of root
*S. f.* HH103 Rif^R^	Wild type surface polysaccharides are produced	7–57^B^×10^4^ (27.7±19.33) C
*S. f.* SVQ530	*S. f.* HH103 *exoA*, EPS is not produced	2–24×10^4^ (11.39±9.40)
*S. f.* SVQ562	*S. f.* HH103 *cgs,* cyclic glucans are not produced	3–30×10^4^ (15.77±13.44)
*S. f.* SVQ536	*S. f.* HH103 *rkpA*, KPS is not produced	6–54×10^4^ (33.00±20.12)
*S. f.* SVQ613	*S. f.* HH103 *lpsB*, LPS is altered	13–15×10^4^ (14.00±1.00)
*S. f.* SVQ581	*S. f.* HH103 *rkpM*, KPS is not produced and LPS is altered.	12–28×10^4^ (18.25±7.14)

A Bibliographic references in which each of the *S. fredii* HH103 SPs mutants is described are listed in Table 6.

B Numbers refer to the highest and lowest values found in the different experiments carried out.

C Mean±standard deviation. At least three independent experiments were carried out for each treatment.

### The *S. fredii* HH103 EPS is involved in biofilm formation

**Figure 8:**
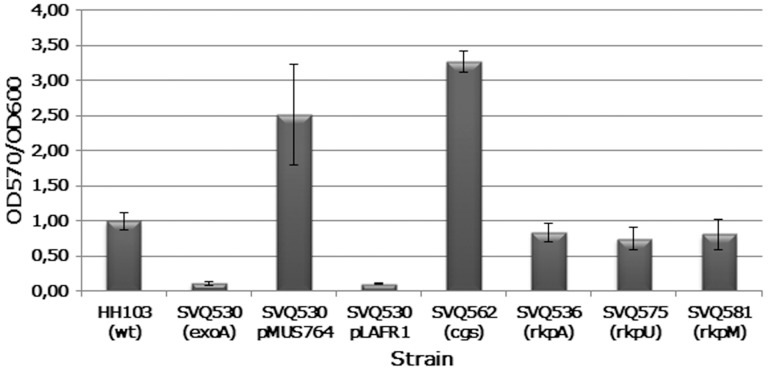
Attachment to plastic surfaces of *S. fredii* HH103 and various mutants affected in surface polysaccharide production. Bacterial biofilm formation was estimated by the relation OD_570_/OD_600_ obtained for the different bacterial cultures. Mutant SVQ530 (*exoA*) complemented with a cosmid clone (pMUS764) carrying the *S. fredii* HH103 *exo* cluster and mutant SVQ530 carrying the empty cosmid vector pLAFR1 were also included in these experiments.

Bacterial surface polysaccharides are known to participate in biofilm formation [8]. Since *S. fredii* HH103 does not bind to the soybean lectin and none of the mutants affected in SPs production are significantly affected in their attachment capacity to soybean roots, we investigated whether any of these polysaccharides is involved in biofilm formation on inert surfaces. *S. fredii* HH103 mutants affected in the production of EPS (mutant SVQ530), cyclic glucans (SVQ562), KPS (SVQ536 and SVQ575), and KPS and LPS (SVQ581) were tested for their capacity to form biofilms on a plastic surface. Mutants SVQ536, SVQ575, and SVQ581 did not show differences with respect to the parental strain HH103 Rif^R^ (Fig. 8).

The biofilm formation capacity of mutant SVQ530 was severely reduced (Fig. 8). Conjugal transfer of cosmid pMUS764 to SVQ530 produced tetracycline-resistant transconjugants that have gained EPS production (data not shown). Cosmid pMUS764 was isolated from a *S. fredii* HH103 genomic library constructed in the cosmid vector pLAFR1 and it restores EPS production in *S. meliloti* AK631, a spontaneous *exoB* mutant of *S. meliloti* Rm41 [50]. Sequencing of the 5′- and 3′-regions of the *S. fredii* HH103 DNA insert cloned in pMUS764 showed that it contains an approximately 25.5-kb segment of plasmid pSfHH103e (covering from 553370 to 578921, accession number HE616899). Thus, with the only exception of *exoP*, cosmid pMUS764 carries all the *exo* cluster of plasmid SfHH103e (Fig. 6). The biofilm formation capacity of mutant SVQ530 carrying pMUS764 was even higher than that of *S. fredii* HH103 Rif^R^ (Fig. 8). This could be due to the fact that the amount of EPS (µg EPS/mL) produced by SVQ530 pMUS764 (85.4±9.6) is significantly higher (α = 5%) than that recovered from HH103 Rif^R^ cultures (68.1±5.7). In fact, mutant SVQ562, which is unable to produce cyclic glucans but overproduces EPS [7], also produced a higher amount of biofilm when compared to its parental wild-type strain HH103 Rif^R^. As expected, introduction of the empty cosmid vector pLAFR1 into SVQ530 did restore neither EPS production (data not shown) nor the capacity to form biofilms (Fig. 8). The growth rates of all strains tested were similar (data not shown).

The putative relevance of *S. fredii* HH103 EPS in bacterial attachment to inert surfaces was further investigated on glass surfaces. Mutants SVQ530 (EPS^-^) and SVQ562 (CG^-^ EPS^++^) were cultivated in MGM medium containing glass slides (see Material and Methods). Optical microscopy of crystal-violet-stained bacteria attached to the slides showed that the *S. fredii* HH103 *exoA* mutant is impaired in its attachment capacity to glass surfaces only at a short incubation period (one day) but not later (four days). In contrast, the attachment capacity of SVQ562 is higher than that of HH103 Rif^R^ at short and long incubation periods (S4 Figure). *S. fredii* HH103 Rif^R^ and its mutant derivative SVQ530 carrying cosmid pMUS764 showed similar capacity to form biofilms on glass surfaces (data not shown).

### *S. fredii* HH103 *cgs* expression is not affected by the *exoA* mutation

*S. fredii* SVQ562 is mutated in the *cgs* gene (formerly called *ndvB*). This mutant fails to produce cyclic glucans but overproduces EPS and its *exoA* gene is transcribed at higher levels than in HH103-Rif^R^ [7]. Two independent real-time reverse-transcription polymerase chain reaction (*rt*-RT-PCR) experiments were carried out to investigate whether the *cgs* gene, encoding a cyclic glucan synthase, was transcribed at higher levels in the *exoA* background (mutant SVQ530) than in the wild type strain HH103-Rif^R^. The expression level of the *cgs* gene in SVQ530 was similar (1.03±0.16) to that present in the wild type strain. Thus, *S. fredii* HH103 *exoA* gene is overexpressed in a mutant unable to produce cyclic glucans (SVQ562) but transcription of the *cgs* gene is not increased in a mutant unable to produce EPS (SVQ530). To our knowledge, this is the first time that *cgs* expression is analysed in an EPS-deficient rhizobial strain. However, previous studies carried out in *S. fredii* NGR234 and *S. meliloti* showed that expression of *cgs* was not affected by changes in osmolarity of the growth medium either [51], [52].

### The *S. fredii* HH103 *exoA* mutant is impaired in surface motility

**Figure 9:**
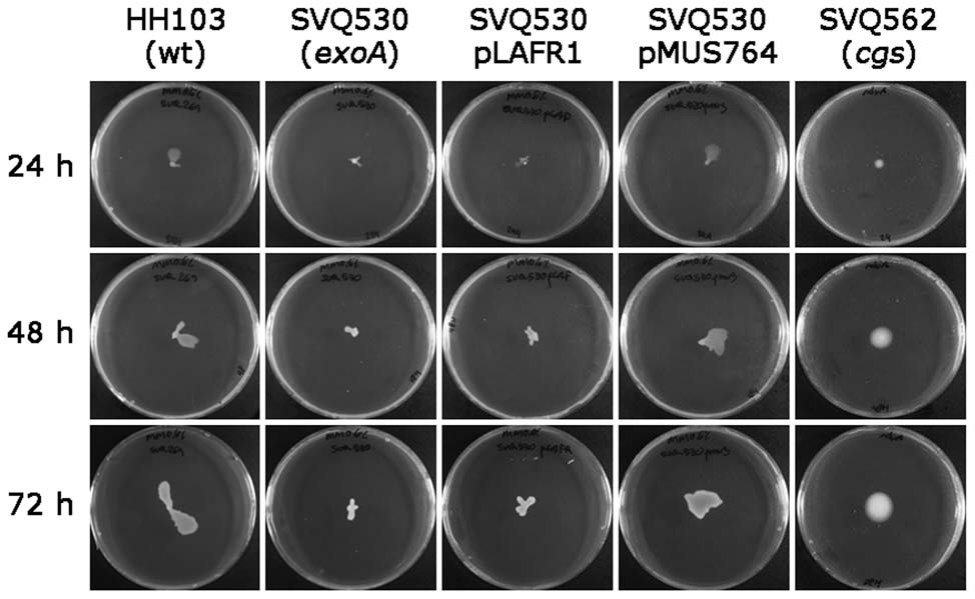
Surface motility of *S. fredii* HH103 Rif^R^, mutant SVQ562 (*cgs*), mutant SVQ530 (*exoA*), mutant SVQ530 complemented with a cosmid clone (pMUS764) carrying the *S. fredii* HH103 *exo* cluster, and mutant SVQ530 carrying the empty cosmid vector pLAFR1 on semisolid MM plates containing 0.6% Difco Agar, Noble (BD). A representative example of at least three experiments is shown.

Recently, the exopolysaccharides EPS I and EPS II produced by *S. meliloti* have been shown to facilitate surface translocation [53], [54]. To investigate if *S. fredii* motility was affected by the *exoA* mutation, strains HH103 and SVQ530 were assayed in motility tests. No significant differences in swimming motility were detected on 0.3% agar BM between the wild type strain (11.4±0.4 mm) and its *exoA* mutant derivative (11.6±0.4 mm). However, a different behavior was observed between these two strains on semisolid MM (0.6% agar). The *exoA* mutation significantly impaired the surface translocation exhibited by the wild type strain, a defect that could be complemented by introducing the *exo* cluster-containing cosmid pMUS764 but not when the empty vector pLAFR1 was used (Fig. 9 and Table 5). The behaviour of the *cgs*-deficient, EPS-overproducing mutant SVQ562 was also examined. This mutant showed a poor surface colonization during the first 24 h but it increased with time. In contrast to the *exoA* mutant, the surface area colonized by SVQ562 after 48 and 72 hours was similar to that of the wild type. These results indicate that *S. fredii* EPS promotes surface translocation. Although colonizing a similar surface area, a different surface spreading pattern was observed for HH103 and SVQ562. Thus, whereas HH103 colonies showed irregular shapes and the presence of protruding tendrils, those formed by SVQ562 were circular with smooth borders. This could indicate that different mechanisms of surface translocation are taken place in these strains but this hypothesis has not been investigated here.

**Table 5:** Surface motility (in mm) of different *S. fredii* strains semisolid MM plates containing 0.6% Difco Agar, Noble (BD).

	24 h	48 h	72 h
**HH103 (wt)**	11.8±0.6 ^a^	13.4±1.2^a^	15.5±1.4^a^
**SVQ530 (** ***exoA*** **)**	8±0.5^b^	9±0.9^b^	8.8±0.6^b^
**SVQ530 pMUS764**	10.1±0.5^a^	13.9±0.9^a^	14.9±1.2^a^
**SVQ562 (** ***cgs*** **)**	6.6±0.1^b^	11.7±0.2^a^	14.5±0.4^a^

Mean values and standard errors obtained from three independent experiments with at least three replicates each.

a, **^b^**Different letters indicate significant differences according to an ANOVA test (P ≤ 0.01).

### Effect of the *exoA* mutation on *S. fredii* HH103 tolerance to osmotic and oxidative stress

To test if the EPS of *S. fredii* HH103 contributes to bacterial protection against unfavourable environmental conditions, tolerances to oxidative and hyperosmotic stresses of mutant SVQ530 were analyzed and compared to those shown by the wild type strain. Differences in sensitivity to the oxidizing agents H_2_O_2_ or paraquat were not detected (data not shown). In contrast, mutant SVQ530 showed an altered tolerance pattern to hyperosmotic stress in comparison to its parental strain. Osmotolerance of *S. fredii* strains was determined in TY and defined MM media supplemented with NaCl (25 mM, 50 mM, 75 mM, 100 mM, 200 mM, 300 mM, and 400 mM) and in MM supplemented with sucrose (5% and 10% W/V).

**Figure 10:**
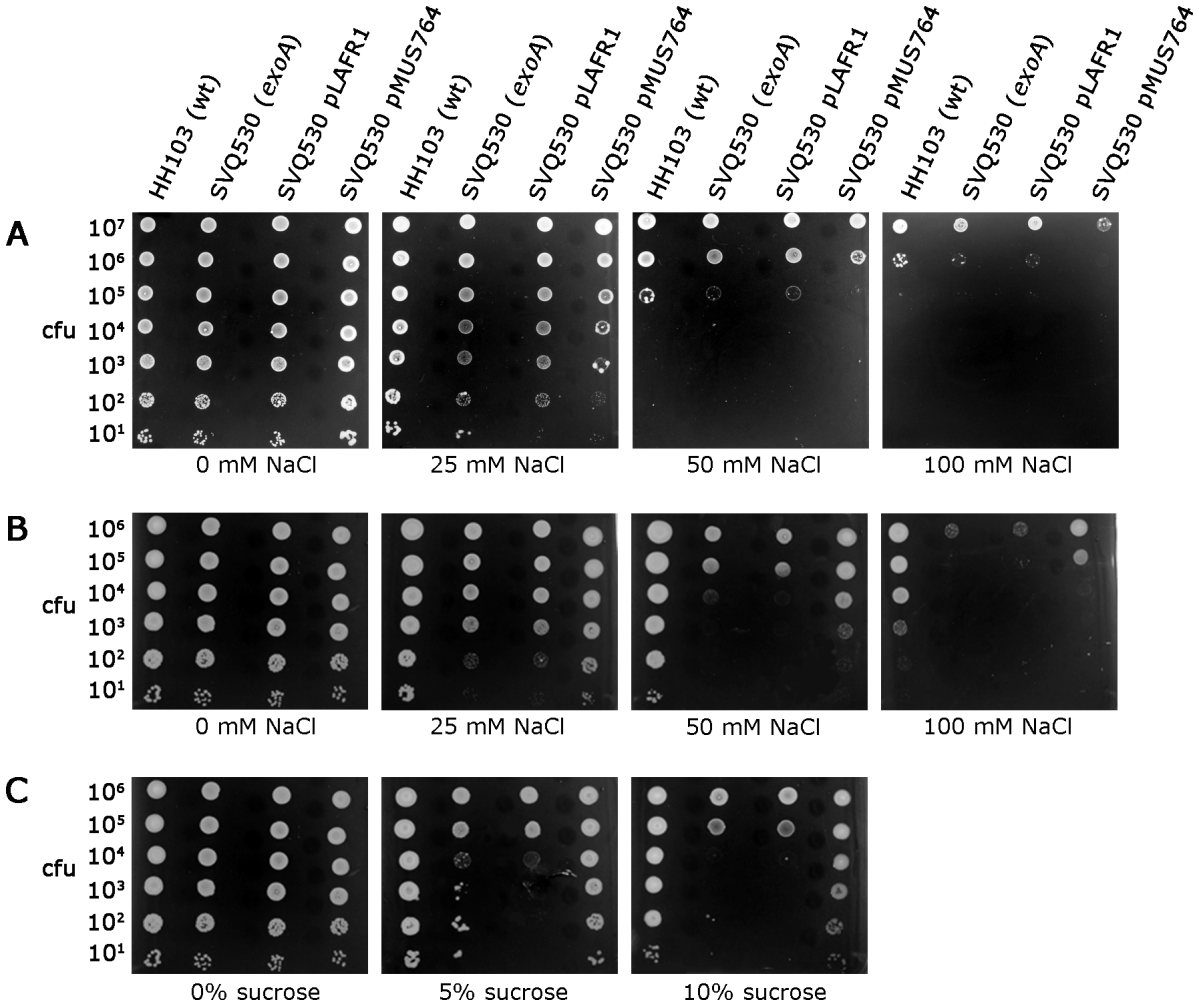
Osmotolerance of *S. fredii* HH103 Rif^R^, mutant SVQ530 (*exoA*), mutant SVQ530 complemented with a cosmid clone (pMUS764) carrying the *S. fredii* HH103 *exo* cluster, and mutant SVQ530 carrying the empty cosmid vector pLAFR1. Bacterial cultures were grown on TY (**A**) and MM (B) supplemented with different concentrations of NaCl, and on MM supplemented with different concentrations of sucrose (**C**). In each row, drops contained approximately the number of CFU indicated on the left. A representative example of at least two experiments is shown. Pictures were taken 5 days after inoculation except for TY medium supplemented with 100 mM NaCl where the picture was taken 7 days after inoculation.

The addition of 50-100 mM of NaCl to TY medium significantly decreased the growth of *S. fredii* HH103 Rif^R^ and its *exoA* mutant derivative, being this effect slightly stronger in the case of SVQ530 (Fig. 10A). On the other hand, SVQ530 (pMUS764) was less osmotolerant in TY medium supplemented with NaCl than HH103 or SVQ530. The fact that the introduction of the empty vector pLAFR1 did not affect SVQ530 survival under this condition suggests that the presence of extra copies of the *exo* cluster could be responsible for the increased osmosensitivity of this strain in TY medium supplemented with NaCl.

The differences in osmotolerance showed by HH103 Rif^R^ and SVQ530 were even stronger in MM media. Thus, whereas the presence of NaCl in MM at a final concentration of 50 mM did not alter the growth ability of the wild type strain, the survival of its *exoA* mutant derivative was severely impaired (Fig. 10B). A higher osmosensitivity of SVQ530 compared to that of the wild type strain was also detected when the osmotic stress was imposed by adding sucrose to MM (Fig. 10C). The presence of cosmid pMUS764, but not that of pLAFR1, increased the survival of SVQ530 in MM media supplemented with sucrose and, partially, in MM media supplemented with NaCl.

## Discussion

*S. fredii* HH103, *B. japonicum*, and *B. elkanii* effectively nodulate many soybean cultivars but produce EPS that are structurally different. *B. japonicum* USDA110 EPS is composed of pentasaccharide subunits containing d-mannose, d-galacturonic acid, d-glucose, and d-galactose in a molar ratio 1∶1∶2∶1 (reviewed by [19]). The EPS produced by sixteen other *B. japonicum* strains has the same sugar composition [55]. The EPS repeating-unit of *B. elkanii* is a tetrasaccharide that contains l-rammnose and d-glucuronic acid in a 3∶1 molar ratio [56]. In contrast, the EPS repeating-unit of *S. fredii* HH103 is a nonasaccharide composed of five units of d-glucose, two units of d-galactose and two units of d-glucuronic acid. Methyl substitutions are present in the slow-growers (*B. japonicum* and *B. elkanii*) while pyruvyl substitutions are found in the fast-grower (*S. fredii*). Acetyl substitutions are present in *B. japonicum* USDA110 and *S. fredii* HH103 but not in *B. elkanii*. In contrast to the structural divergences among the EPS produced by the different soybean-rhizobia analyzed, the *S. fredii* HH103 EPS is more similar to that of *S. meliloti* Rm1021 [16] and identical to the *S. fredii* strain NGR234 EPS [44], two rhizobial strains unable to nodulate soybeans. Hence, the structure of the EPS produced by *S. fredii* HH103 is more related to close-related rhizobia unable to nodulate soybeans than to that of other soybean microsymbionts.

*S. fredii* HH103 *exoA* does not show any symbiotic impairment with *G. max* cv. Williams or *Vigna unguiculata* [11], [38]. The number of nodules formed by soybean and *V. unguiculata* plants inoculated with SVQ530 was slightly higher and slightly lower respectively than in those inoculated with HH103 Rif^R^. Although these differences were not statistically significant, they correlate with the fact that SVQ530 shows enhanced and reduced competitive capacity with soybean Williams and *V. unguiculata* respectively (see Table 2). Thus, the symbiotic importance of *S. fredii* HH103 EPS varies among determinate-nodule-forming legumes, reducing or increasing the bacterial competitive capacity to nodulate.

The apparent lack of specific EPS structural motifs in soybean-nodulating (sino/brady)rhizobia commented above fits well with the general belief that bacterial EPS is not relevant for soybean nodulation. However, an *exoB* mutant of *B. japonicum* USDA110 showed reduced competitivity on soybean [32]. Similarly, other reports have shown that some *B. japonicum* USDA110 EPS mutants were symbiotically defective on *G. soja* PI468397 [33] and PI339871A [57] while here we report that a *S. fredii exoA* mutant formed effective nodules with the wild soybean accession PI597455 and showed increased competitivity on soybean Williams. These differences indicate that the symbiotic roles played by *B. japonicum* and *S. fredii* EPS might be different according to their different structure. This hypothesis is supported by two other results presented here: soybean lectin (SBL) binds to *B. japonicum* but not to *S. fredii* strains (Table 3) and *S. fredii exoA* is not impaired in its attachment capacity to soybean roots. Moreover, the presence of genistein, a flavonoid present in soybean root exudates, provokes a dramatic reduction of the amount of EPS produced in *S. fredii* HH103 [58] but not in *B. japonicum* USDA110 (our own unpublished results).

The total absence of EPS in SVQ530 does not provoke symbiotic impairment with soybeans but increases the bacterial competitive capacity to occupy soybean nodules. *B. japonicum* USDA 110 mutants producing EPS devoid of galactose induced ineffective empty pseudonodules [57] or showed reduced competitiveness for nodulation with *G. max* cv. Preston [32]. The fact that certain alterations of the EPS structure appear to be more deleterious for the symbiosis than the total absence of EPS production suggests that EPS might play a signalling role in early nodulation events and that “wrong signalling” by altered EPS forms could trigger plant defence reactions [32], [33], [59]. A similar situation has been described for another determinate-nodule-forming symbiosis: a *Mesorhizobium loti* R7A *exoA* mutant formed nitrogen-fixing nodules on *Lotus corniculatus* and *L. japonicus* ‘Gifu’ whereas an *exoU* mutant (affected in later steps of EPS biosynthesis) induced empty nodules on both hosts and, occasionally, a few infected nodules following a lengthy delay [60].

Surprisingly, none of the HH103 mutants affected in the production of surface polysaccharides (EPS, KPS, LPS, and CG) used in this study were significantly affected in their attachment capacity to soybean roots (Table 4), suggesting that these polysaccharides might not play a relevant role in the early steps of the soybean-*S. fredii* recognition process. This result is in contrast to the known relevance of cyclic glucans for attachment of other sinorhizobia to their host plants roots, such as that of *S. meliloti* to alfalfa [61] or that of *S. fredii* NGR234 to *Vigna unguiculata* and *Leucaena leucocephala* roots [51]. Moreover, our results also indicate that the molecular mechanisms mediating in *S. fredii* HH103 attachment to soybean roots could be different from that operating in the *B. japonicum*-soybean symbiosis. One could hypothesise that S. *fredii* HH103 adhesins might act as relevant components in bacterial attachment to soybean roots while the interaction between *B. japonicum* EPS and soybean lectins could be pivotal in the first steps of this interaction. In the *R. leguminosarum*-*Pisum sativum* symbiosis [62] it has been established that under slightly alkaline conditions, bacterial attachment would mainly take place through a rhicadhesin-mediated mechanism whereas under acidic conditions plant lectins would be essential for bacterial attachment to the plant [62], [63]. In accordance to this hypothesis, several reports indicate that *S. fredii* is more competitive than *B. japonicum* if soybeans are growing under alkaline conditions while the former is overcompeted by the latter under acidic conditions [64], [65].

Results presented here indicate that neither HH103 mutants affected in KPS (SVQ536) nor those affected in both KPS and LPS (SVQ575 and SVQ581) are affected in their attachment capacity to plastic surfaces (Fig. 8). *S. fredii* HH103 mutants affected in LPS (*lpsB* and *lpsE*) formed biofilms that were similar to those produced by HH103 Rif^R^ [10]. Thus, in *S. fredii* HH103, neither KPS nor LPS are relevant for this bacterial trait. In contrast, the absence of EPS (*exoA* mutant) reduced dramatically the amount of biofilm formed on plastic surfaces. Introduction of cosmid pMUS764, carrying *exoA* as well as most of the HH103 *exo* genes, not only restored EPS production but also led to increased biofilm formation. In fact, the amount of EPS produced by SVQ530 pMUS764 is slightly, but significantly, higher than that formed by HH103 Rif^R^. Interestingly a HH103 *cgs* mutant (SVQ562), unable to produce CG, overproduces EPS [7] and also forms a higher amount of biofilm on plastic (Fig. 8) and glass (S4 Figure) surfaces when compared to HH103. Similarly, *Pseudomonas fluorescens* hypermucoid mutants show increased biofilm formation [66]. However, a *S. meliloti exoS* mutant overproduces EPS and forms a thicker but less stable biofilm [67], which indicates that EPS overproduction does not necessarily correlate with increased biofilm formation.

Bacterial motility is an important factor for the establishment of symbiosis under natural soil conditions. In *S. meliloti* several regulatory proteins, such as MucR, ExoR, and ExpR, are involved in both EPS production and flagella-driven motility (reviewed by [19]). In addition, it has recently been reported an EPS-mediated sliding motility in *S. meliloti* [53]. The impairment in surface spreading shown by the EPS deficient *exoA* derivative mutant of *S. fredii* HH103 indicates that EPS promotes surface motility in this rhizobium. The EPS overproduction characteristic of the *cgs* mutant (SVQ562) could account for the similar surface colonization efficiency exhibited by this strain compared to the wild type, although this has not been demonstrated in this work. The different macroscopic appearance of HH103 and SVQ562 colonies on semisolid surfaces could be an indication that different mechanisms are involved. Considering that the *cgs* mutant is impaired in flagellar motility [7], one can speculate that the surface spreading shown by this strain is an EPS-mediated sliding motility similar to that described for *S. meliloti*.

One of the structural functions assigned to EPS produced by rhizobia is to act as a protective barrier against environmental stresses and antimicrobial compounds [8], [19], [68]. In fact, a positive correlation between increased EPS production and enhanced protection against H_2_O_2_ has been demonstrated in *Azorhizobium caulinodans* [69]. EPS production has also been positively correlated with desiccation tolerance in *R. leguminosarum* [70] and hyperosmotic stress has been found to induce up-regulation of *exo* genes in *S. meliloti* [52]. Results presented here demonstrate that the *S. fredii* SVQ530 mutant is more sensitive to hyperosmotic stress conferred by both ionic (e.g. NaCl) and non-ionic (e. g. sucrose) osmolites than its parental wild-type strain HH103 Rif^R^ (Fig. 10). The differences observed are higher in MM than in TY, which correlated with the fact that bacterial mucosity is reduced in the latter (in fact TY was formulated with the only purpose of reducing bacterial mucosity). Cosmid pMUS764, which carries most genes of the *exo* cluster, not only restored the mucoid phenotype of SVQ530 but also led to an increased production of EPS with regard to the parental strain HH103. Interestingly, the presence of this cosmid had a positive and a negative effect on SVQ530 osmotolerance to NaCl in MM and TY media respectively, suggesting that in the latter medium a correct balance in the production of EPS is important for appropriate growth upon ionic hyperosmotic stress. This hypothesis might also explain why the ability of cosmid pMUS764 to complement SVQ530 growth in MM medium is better under non-ionic (sucrose) than under ionic (NaCl) hyperosmotic stress.

In summary, the results presented in this work indicate that bacterial EPS is dispensable in the *S. fredii* HH103-soybean symbiosis. Although this polysaccharide is important for bacterial surface motility and biofilm formation on abiotic surfaces and increases bacterial osmotolerance, it is not involved in root attachment. The fact that the absence of EPS increases competitiveness capacity with soybean could explain why the production of this polysaccharide is downregulated by genistein.

## Materials and Methods

### Isolation and purification of the *S. fredii* HH103 EPS

Culture medium (1.2 L) was treated with proteinase K (10 µg/mL, 6 hours a 37°C). Then, it was concentrated up to 20% of the initial volume on a rotary evaporator and three volumes of ethanol were added. After 24 h at 4°C, the resulting precipitate was separated by centrifugation, re-dissolved in water and purified by dialysis against distilled water at 4°C. The solution was concentrated and freeze dried, yielding 404 mg of exopolysaccharide.

### Monosaccharide analysis

Monosaccharides were identified on Gas-liquid chromatography coupled with mass spectrometry (GLC-MS) separation of their per-*O*-trimethylsilylated methyl glycosides [71]. The absolute configurations of monosaccharides were assigned following GLC-MS analysis of their per-*O*-trimethylsilylated (*S*)- and (*R,S*)-2-butyl glycosides as described by Gerwig et al. [72]. Derivatives from standard monosaccharides were prepared for comparison. GLC-MS was performed on an Agilent Technologies GC system 7890A coupled to a mass spectrometer 5975C fitted with a column HP-5MS (30 m×0.25 mm, Agilent). The temperature program for separating the per-*O*-trimethylsilylated methyl glycosides was isothermal at 140°C for 2 min, followed by an 8°C/min gradient up to 280°C, and isothermal for 10 min. The temperature program for separating the per-*O*-trimethylsilylated 2-butyl glycosides was isothermal at 130°C for 3 min, followed by a 3°C/min gradient up to 150°C, then increasing at 10°C/min up to 250°C, then isothermal for 30 min. The ionization potential was 70 eV, and spectra were recorded in low-resolution mode.

### Reduction of carboxylic groups

Carboxylic groups were reduced to deuterated hydroxymethyl groups (-CD_2_OH) by the method of carbodiimide as described in Rodríguez-Carvajal et al. [73].

### Methylation analysis

Vacuum-desiccated samples of polysaccharide having carboxyl groups previously reduced were methylated by using the method of Ciucanu and Costello [74]. Hydrolysis, reduction with NaBD_4_, and acetylation was performed according to Kim et al. [75], yielding the corresponding partially methylated and acetylated alditols (PMAAs), which were solubilized in 80 µL of CH_2_Cl_2_, and analyzed by GLC-MS. Gas–liquid chromatography–mass spectrometry was performed on a Hewlett-Packard 5890 Series II instrument fitted with a TRB-1 column (25 m×0.25 mm, Teknokroma) coupled to a 5971 Series mass spectrometer. The temperature program was isothermal at 120°C for 1 min, followed by an 8°C/min gradient up to 250°C, and isothermal for 30 min. The ionization potential was 70 eV, and spectra were recorded in low-resolution mode. Peak assignments were made based on retention times and mass spectra against PMAA standards [76].

### Partial hydrolysis assays

A solution of the exopolysaccharide (250 mg) in 0.5 M TFA (40 mL) was heated for 1 h at 100°C, and then dialysed against water. The diffusate was concentrated and the residue was chromatographed on Bio-Gel P-2 using water as eluent. The effluent was monitored by thin layer chromatography and fractionated accordingly. The non-diffusable fraction was lyophilized, and the procedure was repeated. Silica gel was added to fractions containing low molecular-weight oligosaccharides and the suspensions were freeze dried. The powder was deposited at the top of a silica gel column (1×0.5 cm), and a gradient (acetonitrile, then acetonitrile/water 95∶5, 90∶10, 85∶15, and 75∶25 (v/v)) was used to elute the oligosaccharides [77].

### Lithium degradation analyses

The polysaccharide was stirred in ethylenediamine with lithium metal as described in Fernández de Córdoba et al. [78], and then purified by size exclusion chromatography on Biogel P-2.

### NMR experiments

Samples were deuterium-exchanged several times by freeze-drying from D_2_O and then examined in solution (1–5 mg/750 µL) in 99.98% D_2_O. Spectra were recorded at 303 K and 333 K on a Bruker AV500 spectrometer operating at 500.20 MHz (^1^H) and 125.8 MHz (^13^C). Chemical shifts are given in ppm, using the HDO signal (4.71 ppm at 303 K, 4.40 ppm at 333 K) (^1^H) as reference. Standard Bruker sequences were used for 2D experiments [79]. The TOCSY experiment was acquired using a data matrix of 256×2K points to digitize spectral widths of 3930 to 5081 Hz; an isotropic mixing time of 80 ms was used.

The 2D homonuclear DQF-COSY was performed by using a data matrix of 256×1K points to digitize a spectral width of 3930 to 5081 Hz. The 2D heteronuclear one-bond proton-carbon correlation experiment was registered in the ^1^H-detection mode via single-quantum coherence (HSQC). A data matrix of 256×1K points was used to digitize a spectral width of 4006 to 5681 in F2 and 22522 Hz in F1. ^13^C decoupling was achieved by the GARP scheme. Squared-sine-bell functions were applied in both dimensions, and zero-filling was used to expand the data to 1K×1K. The HMBC experiment was performed using the Bruker standard sequence with 256 increments of 1K real points to digitize spectral widths of 4006 to 7334 Hz in F2 and 30030 Hz in F1; a delay corresponding to 8 Hz was used for evolution of long-range couplings. The ROESY experiment was performed with a mixing time of 100 ms. A data matrix of 256×2K points was used to digitize a spectral width of 5681 Hz; 32 scans were used per increment. HMQC-TOCSY was acquired using a data matrix of 256×1K points to digitize spectral widths of 4807 and 22525 Hz in F2 and F1, respectively. NMR spectra have been assigned using the program Sparky [80].

### Microbiological and Molecular techniques

The bacterial strains and plasmids used in this work are listed in Table 6. *Sinorhizobium fredii* strains were grown at 28°C on TY medium [81], yeast extract/mannitol (YM) medium [82], MGM medium [83], Bromfield medium (BM) (0.04% tryptone, 0.01% yeast extract, and 0.01% CaCl_2_·2H_2_O) or in minimal medium (MM) containing glutamate (6.5 mM), mannitol (55 mM), mineral salts (K_2_HPO_4_, 1.3 mM; KH_2_PO_4_·3H_2_O, 2.2 mM; MgSO_4_·7H_2_O, 0.6 mM; CaCl_2_·2H_2_O, 0.34 mM; FeCl_3_·6H_2_O, 0.022 mM; NaCl, 0.86 mM), biotin (0.2 mg/L) and calcium pantothenate (0.1 mg/L) [84]. *Escherichia coli* was cultured on Luria-Bertani (LB) medium [85] at 37°C. When required, the media were supplemented with the appropriate antibiotics as described by Vinardell et al. [49]. Osmotic stress was achieved by adding different concentrations of either NaCl (25 mM, 50 mM, 75 mM, 100 mM, 200 mM, 300 mM, 400 mM) or sucrose (5, 10 and 15% w/v) to the indicated media. Flavonoids were dissolved in ethanol at a concentration of 1 mg/mL and used at 1 µg/mL. Plasmids were transferred from *E. coli* to rhizobia by triparental mating by using pRK2013 as the helper plasmid [86]. The Optical Density (OD) of bacterial cultures was determined by using a Pharmacia LKB Novaspec II spectrophotometer.

**Table 6:** Bacterial strains and plasmids.

Strain or plasmid	Derivation and relevant properties	Source or reference
***Sinorhizobium fredii***		
HH103	Wild type strain	[34]
SVQ269	HH103-Rif^R^	[88]
SVQ530	SVQ269 *exoA*::*lacZ*-Gm^r^	[11]
SVQ536	SVQ269 *rkpA*::*lacZ*-Gm^r^	[33]
SVQ562	SVQ269 *cgs*::*lacZ*-Gm^r^	[7]
SVQ575	SVQ269 *rkpU*::Ω	[38]
SVQ581	SVQ269 *rkpM*::Ω	[39]
SVQ613	SVQ269 *lpsB*::Ω	[10]
USDA192	Wild-type strain	[92]
B1, B2, B4, B6, B8, B33, B50	Wild type strains from soil samples of Xingjian province	[93]
S1, S2, S4, S5, S8, S28, S44, S48, S49	Wild type strains from soil samples of Hubei province	[93]
HH3, HH4, HH5, HH18, HH25, HH29, HHG35, WH4, WHG11 WHG14-S, WH5, WH7	Wild type strains from soil samples of Henan province	[93]
HW1, HW5, HW16, HW22, HWG35, WW2, WW9, WWG11, WWG14	Wild type strains from soil samples of Shan Dong province	[93]
NGR234	Broad host range strain isolated from nodules of *Lablab purpureus*	[94]
***Bradyrhizobium japonicum*** USDA6, USDA38, USDA110, USDA123, USDA122, USDA136 ( = CB1890), USDA138	Wild type strains	USDA^A^
***Bradyrhizobium elkanii*** USDA6 and USDA76	Wild type strains	USDA
***Bradyrhizobium liaoningense*** 2281	Wild type strain	[95]
***Bradyrhizobium pachyrhizi*** PAC48	Wild type strain isolated from *Pachyrhizus ahipa*	[96]
***Bradyrhizobium canariense*** ISLU16	Wild type strain isolated from *Ornithopus compressus*	[97]
**Plasmids**		
pLAFR1	Cosmid vector, Tc^R^	[98]
pMUS764	pLAFR1 carrying the *exo* region of the symbiotic plasmid pSfHH103e	This work
pRK2013	Helper Plasmid, Km^R^	[99]

A USDA: U.S. Department of Agriculture, Beltsville, MD (USA).

For EPS quantification, bacterial cultures were grown on YM for 96 hours at 28°C (OD_600_ = 1.2–1.3) under shaking conditions. Cells were removed by centrifugation (20000 *g*, 15 min) and total carbohydrate amounts of the EPS-containing supernatants were determined using the anthrone-H_2_SO_4_ method, which measures the total reducing sugar content in a given sample, as previously described [87]. Four independent experiments in duplicate were carried out. The amounts of EPS produced by each strain were compared by the nonparametric test of Kruskal-Wallis.

### Nodulation and competition for nodulation assays

Nodulation assays of *Glycine max* cv. Williams (soybean) and *Vigna unguiculata* cv. Bisbee Red (cowpea) were carried out as described by Hidalgo et al. [38]. Competition for nodulation between *S. fredii* SVQ530 and its parental strain HH103 Rif^R^ were performed as previously described [88]. Nodule occupancy by strain SVQ530 was determined by assessing the gentamycin resistant marker (presence of the *lacZ*Δp-Gm^R^ cassette) of at least one hundred isolates from soybean or cowpea nodules. Determinations were carried out 6 weeks after inoculation.

### Soybean lectin (SBL) binding assays

Fourteen bradyrhizobia and 42 *S. fredii* strains grown on nitrocellulose filters were tested for soybean lectin (SBL) binding capacity. Binding assays using peroxidase-labelled soybean lectin (Sigma) were carried out as described by Liang and Emerich [48]. Positive SBL binding was scored when any particular strain developed a dark blue colour spot on the growth area of the filter after immersion in development solution (60 mg of chloronaphthol, 15 mL of methanol, 85 mL of PBS, and 50 µL of a 30% hydrogen peroxide) for 10 min at room temperature.

### Bacterial attachment to roots

Surface disinfected and pre-germinated soybean seeds cv. Williams were sown in sterile glass containers of 200 mL filled up with quartz sand, watered with a nutrient solution [89], one seed per container. Plants were placed in a growth chamber (16 h at 25°C/8 h 18°C day/night, and 88% RH). Roots were harvested 5 days after sown, washed up with sterile water to eliminate most of the adhered sand, aseptically weighted, and then transferred to sterile 15 mL plastic tubes. Roots were incubated with 30 mL of each bacterial culture (10^5^–10^6^ bacteria/mL) during 4 h in a rotary shaker, at 120 rpm. The procedure for the estimation of the viable cells attached to roots basically followed Albareda et al. [90] methodology, except that the whole root system was used and that a blender (IKA Ultra-Turrax® tube drive) was used to obtain root homogenates.

### Motility and biofilm formation assays

Swimming was examined on plates prepared with BM containing 0.3% Bacto agar and inoculated with 3 µL droplets of rhizobial cultures grown in TY (OD_600nm_ = 1). Surface motility was analyzed on semisolid MM plates containing 0.6% Difco Agar, Noble (BD) by inoculating 2 µL of washed 10-fold concentrated cultures grown in TY broth to the late exponential phase. The migration zone was determined as the colony diameter (mm) after 2 days (for swimming motility), or 24, 48 and 72 hours (for surface motility on semisolid MM) of incubation.

Assays for biofilm formation on plastic surfaces were carried out as described by Margaret et al. [10]. Cultures were grown in MGM medium as described above, diluted to an OD_600nm_ of 0.2, and inoculated into the microtiter polystyrene plate wells in 100-µL aliquots. The plates were covered with a sterile lid to prevent evaporation, turned upside down and incubated without agitation at 28°C for 48 h. Cell growth was quantified by measuring the OD_600nm_ at the end of the experiment. The contents of each well were then removed, the wells were dried at room temperature and washed three times with 100 µL of sterile physiological saline solution in order to remove all non-adherent bacteria. The plates were emptied, air dried, stained for 20 min with 100 µL of 0.1% crystal violet per well, air dried, then rinsed three times with water and air dried. Biofilm formation was quantified by the addition of 100 µL of 96% ethanol to each crystal violet-stained microtiter dish well, and the OD_570nm_ of the solubilized crystal violet was determined with a microplate reader (Synergy HT; BioTek; Winooski, Vermont, USA). Data presented are the media of at least three independent experiments performed in duplicate; in each experiment, at least 12 wells for each treatment were measured.

In order to visualise biofilm formation on glass surface, *S. fredii* strains were cultured in MGM medium (28°C, static conditions) in sterile Glass Coplin Staining Jars containing glass slides. 1 and 4 days after inoculation glass slides were collected, air dried, stained with 0.1% crystal violet (1 min), rinsed with water, air dried, and observed and photographed using an Olympus BX61 optic microscope.

### Expression of the *cgs* gene in a *S. fredii* HH103 *exoA* mutant

For RNA extraction, *S. fredii* strains were incubated in YM broth in an orbital shaker (180 rpm) at 28°C. When the cultures reached an OD_600nm_ of 0.4–0.5, cells were harvested, and RNA was extracted by using the RNAprotect Bacteria Reagent, the RNAeasy mini kit, and the RNase-free DNase Set (all provided by Qiagen, Basel, Switzerland) following the manufacturer's instructions. To ensure that genomic DNA was not present in RNA samples, a control PCR was performed employing primers q*ndvB*-F (5′ actattctctttcactgg) and q*ndvB*-R (5′ gcgttgcttctgactatg). Only those RNA samples showing no PCR amplification were selected for further work. Reverse transcription of total RNA was carried out using the Quantitect kit (Qiagen), which includes a genomic DNA elimination step before RNA reverse transcription.

*q*PCR experiments were performed in a 20-µL final volume containing 1 µL of cDNA, 0.6 pmol of each primer, and 10 µL of FastStart SYBR Green Master Mix (Roche Diagnostics). PCR was conducted on the iCycler IQ (Bio-Rad Laboratories SA, Marnes La Coquette, France), and the threshold cycles were determined with the iCycler software. Primers used for amplification of a 112-bp internal fragment of the *S. fredii* HH103 *cgs* cDNA were q*ndvB*-F and q*ndvB*-R. To normalize the data, a 197-bp internal fragment of the *S. fredii* HH103 16S rRNA (accession number AY260145) was employed as an internal control in each sample by using primers HH16S-F and HH16S-R [10]. For each strain the relative expression of *cgs* with regard to that of HH103 was calculated by using the formula 2^ΔΔCT^, where **ΔΔ**C_T_  =  (C_T *cgs*_ - C_T 16S_) problem strain - (C_T *cgs*_- C_T 16S_) HH103. Two independent experiments performed in triplicate were carried out and averaged. These two experiments were performed with different, independently extracted RNA samples for each strain.

### Hydrogen peroxide and paraquat sensitivity tests

Sensitivity to H_2_O_2_ and paraquat was determined by a disc diffusion assay performed basically as previously described [91] with some modifications. Briefly, *S. fredii* cells were grown from glycerol stocks to mid logarithmic phase (OD_600nm_ = 0.5) in TY broth supplemented with the corresponding antibiotics. For the filter disk assay, 50 µL of culture was added to 3 mL TY of soft agar (7 g/L) and poured onto TY plates. After 30 min, a paper disk (6 mm diameter) previously soaked with 5 µL of the agent to be tested (1 M H_2_O_2_ or 1 M paraquat was placed in the center of the plate. At least three plates were prepared for each agent tested. Plates were incubated for 48 h at 30°C, and then the diameter of growth inhibition was recorded.

## Supporting Information

S1 Figure **^1^H (500 MHz)-^13^C (125 MHz) HSQC of the trisaccharide isolated from partial hydrolysis of EPS.** Primes indicate that the residue is not the same as found in the polysaccharide.
(TIF)

S2 Figure **^1^H (500 MHz, 353 K) of anomeric and acetyl regions of EPS a) Initial; b) After several hours of heating.**
(TIF)

S3 Figure **Plant responses to inoculation of** ***Glycine max*** **cv. Williams (panels A, B, E) and** ***Vigna unguiculata*** **cv. Brisbee (panels C, D, F) with** ***Sinorhizobium fredii*** **HH103 Rif^R^ (A, C, E, F) and its** ***exoA*** **mutant derivative (B, D, E, F).** Panels A to D show nitrogen-fixing root nodules. Panels E and F show plant aerial parts.
(TIF)

S4 Figure **Attachment to glass surfaces of** ***S. fredii*** **HH103 Rif^R^, and its** ***exoA*** **(SVQ530) and** ***cgs*** **(SVQ562) mutant derivatives.** Bacterial cultures were grown in MGM medium for one (1 dpi) and four days (4 dpi), then glass slides were stained with crystal violet and visualised in the microscope at 200X and 1000X.
(TIF)

S1 Table **Chemical shifts (^1^H and ^13^C) for the lithium-degraded polysaccharide obtained from EPS.**
(DOCX)
