# Supplementary figures and images for: Structure and Biological Roles of Sinorhizobium fredii HH103 Exopolysaccharide
Source: PLoS One. 2014 Dec 18;9(12):e115391. doi: 10.1371/journal.pone.0115391 (PMC4270759; doi:10.1371/journal.pone.0115391)

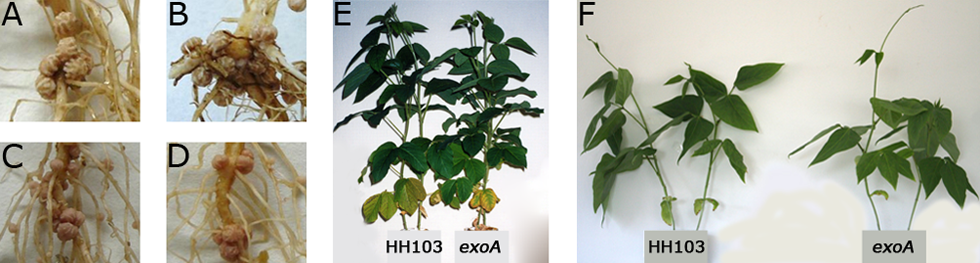

Supplement: S3 Figure — Plant responses to inoculation of Glycine max cv. Williams (panels A, B, E) and Vigna unguiculata cv. Brisbee (panels C, D, F) with Sinorhizobium fredii HH103 RifR (A, C, E, F) and its exoA mutant derivative (B, D, E, F). Panels A to D show nitrogen-fixing root nodules. Panels E and F show plant aerial parts. (TIF) [file pone.0115391.s003.tif]

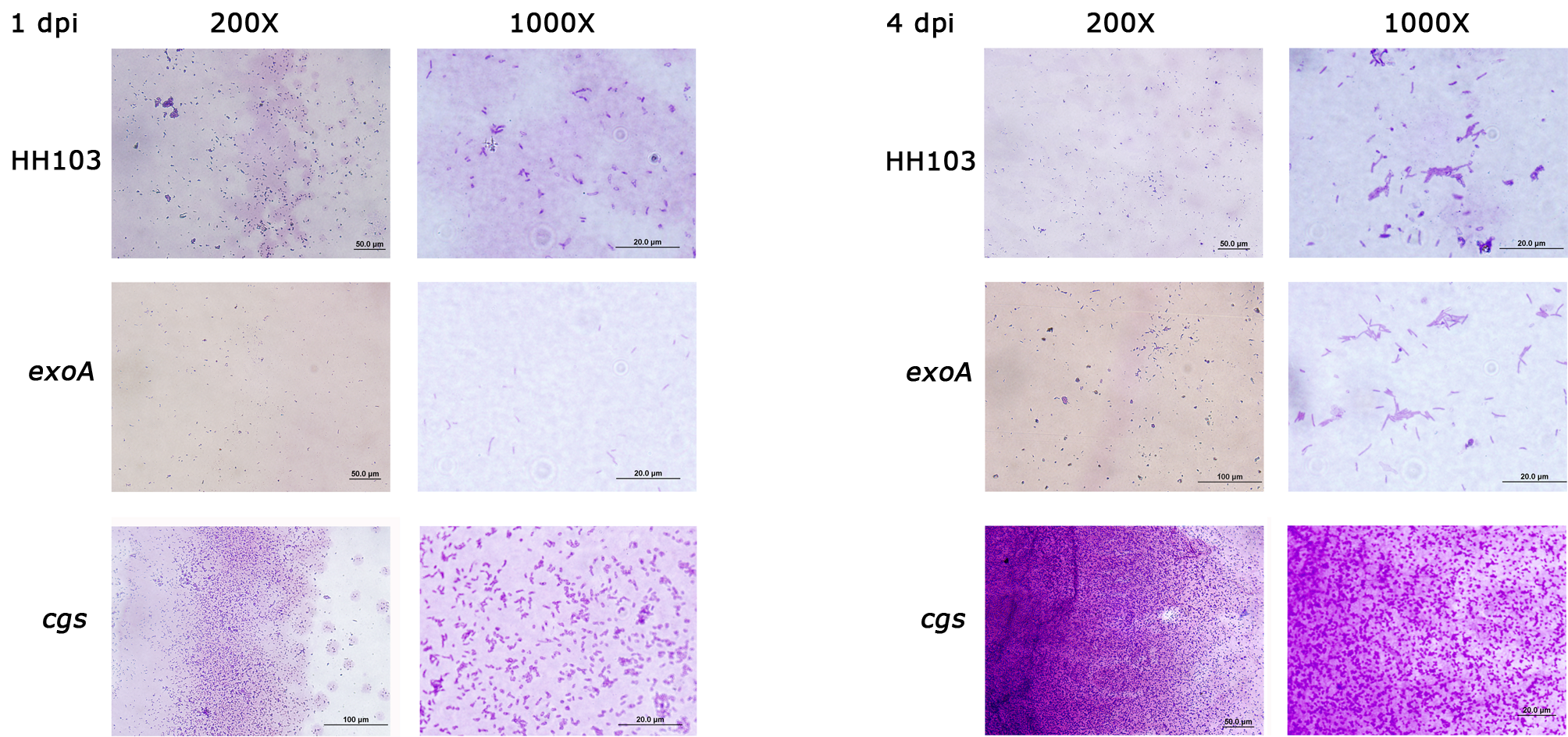

Supplement: S4 Figure — Attachment to glass surfaces of S. fredii HH103 RifR, and its exoA (SVQ530) and cgs (SVQ562) mutant derivatives. Bacterial cultures were grown in MGM medium for one (1 dpi) and four days (4 dpi), then glass slides were stained with crystal violet and visualised in the microscope at 200X and 1000X. (TIF) [file pone.0115391.s004.tif]
