# Supplementary material for: Structure and Biological Roles of Sinorhizobium fredii HH103 Exopolysaccharide
Source: PLoS One. 2014 Dec 18;9(12):e115391. doi: 10.1371/journal.pone.0115391 (PMC4270759; doi:10.1371/journal.pone.0115391)
Supplement: S1 Table — Chemical shifts (1H and 13C) for the lithium-degraded polysaccharide obtained from EPS. (DOCX) [file pone.0115391.s005.docx]

**Table S1.** Chemical shifts (^1^H and ^13^C) for the lithium-degraded polysaccharide obtained from EPS.

| **Unit** | **Signal** | **1** | **2** | **3** | **4** | **5** | **6a** | **6b** |
| --- | --- | --- | --- | --- | --- | --- | --- | --- |
| A | ^1^H | 4.5 | 3.4 | 3.5 | 3.7 | 3.6 | n.a **^a^** | n.a |
| →4)-β-d-Glc*p* | ^13^C | 103.1 | 73.5 | 76.2 | 79.3 | 75.3 | n.a |  |
| B | ^1^H | 4.51 | 3.34 | 3.46 | 3.42 | 3.78 | 4.29 | 3.94 |
| →6)-β-d-Glc*p* | ^13^C | 103.2 | 73.6 | 76.3 | 70.3 | n.a | 68.7 |  |
| C | ^1^H | 4.54 | 3.34 | 3.51 | 3.50 | 3.62 | 4.19 | 3.89 |
| →6)-β-d-Glc*p* | ^13^C | 103.2 | 73.6 | 76.1 | 70.1 | 75.3 | 69.2 |  |
| D | ^1^H | 4.55 | 3.38 | 3.79 | 3.76 | n.a | n.a | n.a |
| →4)-β-d-Glc*p* | ^13^C | 102.8 | 73.5 | 74.1 | 78.7 | n.a | n.a |  |
| E | ^1^H | 4.71 | 3.43 | 3.67 | 3.67 | 3.60 | 3.97 | 3.83 |
| →4)-β-d-Glc*p* | ^13^C | 103.9 | 73.6 | 74.8 | 79.2 | 75.1 | 60.6 |  |
| F | ^1^H | 4.53 | 3.72 | 3.83 | 4.17 | 3.73 **^b^** | 3.78 | 3.78 |
| →3)-β-d-Gal*p* | ^13^C | 103.0 | 70.6 | 82.4 | 68.8 | 75.4^b^ | 61.4 |  |

**^a^** Not assigned.

**^b^** Assignment based on literature data.
